# Prevalence and Associated Factors of Unprotected Anal Intercourse with Regular Male Sex Partners among HIV Negative Men Who Have Sex with Men in China: A Cross-Sectional Survey

**DOI:** 10.1371/journal.pone.0119977

**Published:** 2015-03-27

**Authors:** Dongliang Li, Chunrong Li, Zixin Wang, Joseph T. F. Lau

**Affiliations:** 1 Chaoyang Center for Disease Control and Prevention, Beijing, China; 2 Centre for Health Behaviours Research, JC School of Public Health and Primary Care, The Chinese University of Hong Kong, Hong Kong SAR, China; 3 CUHK Shenzhen Research Institute (SZRI), Shenzhen, China; 4 Centre for Medical Anthropology and Behavioral Health, Sun Yat-sen University, China; UCSF, UNITED STATES

## Abstract

The HIV prevalence and incidence among men who have sex with men (MSM) in China are high. Unprotected anal intercourse (UAI) with a regular male sex partner (RP), a significant predictor of HIV sero-conversion, was high yet under-emphasized among MSM having RP (MSMRP). The present cross-sectional survey interviewed 307 HIV negative MSMRP recruited through convenient sampling from multiple sources, including venue-based outreaching, online recruitment, and referrals made by peers, in Beijing and Chengdu, China. Among MSMRP, the prevalence of UAI with RP in the last three months was 52.4%. The results of the multivariate analysis showed that trust and intimacy within the relationship with RP and presence of clinical depression symptoms were positively associated with UAI with RP in the last three months. Other associated scalar factors derived from the Theory of Planned Behavior were related to perceptions on condom use, including positive attitudes toward condom use (a negative association), subjective norm of the perception that MSM do not usually use condoms during anal intercourse with RP (a positive association), perceived behavioral control over condom use with RP (a negative association), and behavioral intention to use condoms with RP in the coming three months (a negative association). It is seen that MSMRP were at high risk of HIV/STD transmission. The associated factors hence involved those related to perceptions about condom use, mental health, and interpersonal relationship. Future interventions should take these multi-dimensional factors into account. In particular, future research to test the efficacy of couple-based interventions that include mental health elements needs to be conducted, as trust and intimacy within the relationship were associated with UAI among MSMRP, and mental health problems may exist for both the MSMRP and their RP.

## Introduction

HIV prevalence among men who have sex with men (MSM) has increased sharply in China. Meta-analysis reported HIV prevalence of 5.3% among MSM in China (2009) [1], and prevalence was higher than 20% in provinces such as Guizhou and Chongqing [2,3]. HIV prevalence has also increased at a very high speed in many Chinese cities over the last decade [4–6]. For instance, it increased from 0.6% in 2004 [4] to 13.1% in 2011 among MSM in Beijing [5] and from 1.06% in 2004 to >15% in 2010 in Chengdu [6]. HIV incidence, a measure of newly diagnosed cases among MSM in China, was also exceptionally high, ranging from 4.17 to 8.09 per 100 person-years in 2009 in Nanjing, Shenyang, and Beijing [7–9].

In Western countries, there is a concern about HIV transmission among MSM who are in regular partnerships [10]. Regular male sex partners (RP) of MSM are commonly defined as “boyfriends”, or those who are in a stable relationship that does not involve transactional sex [11]. Over half of the MSM have RP (MSMRP) in Switzerland [12] and in London [13]. In China, the prevalence of MSMRP among MSM ranged from 47.2% to 62.1% [14–16]. However, regular partnership does not always imply monogamy. Indeed slightly over half of MSMRP have had sexual intercourse outside the regular partnership in countries such as the U.K. [13], the U.S. [17] and in some parts of China (e.g. Nanjing) [18].

Multiple male sex partnership speeds up HIV transmission. It is a known risk factor of HIV sero-conversion [19,20]. Unprotected anal intercourse (UAI) among MSM was more likely to occur when RP was involved, as shown by results of previous studies that were conducted in both western countries [21–23] and in China [13,24]. Furthermore, UAI with RP, which was highly prevalent (42.9% to 78.1%) among MSMRP in China [25–27], was a significant predictor of HIV sero-conversion among MSM according to a cohort study conducted in Nanjing, China [26]. It is therefore a public health concern to understand factors associated with UAI with RP among MSMRP. Such factors are multi-dimensional. Interpersonal-level factors are defined as features or characteristics of the relationship between MSM and their male sex partners (e.g., duration of the relationship, trust and intimacy within the relationship) [28–37], while individual-level factors refer to participants’ personality, knowledge, cognitions, and mental health status [23–39].

A number of qualitative studies explored the significance of inter-personal factors in explaining risk behaviors among MSMRP. It was found that the duration of regular partnership and familiarity with the RP were positively associated with perception of low possibility of being harmed by the RP, which was in turn associated with UAI with the RP among MSM [29]. Trust may also lead to risky sex behaviors among MSMRP [28,29,30], as MSMRP assume that their trusted RP would be frank about their sexual experiences and become convinced that the RP is risk-free [31]. Similarly, MSMRP would perceive a low level of risk associated with UAI with their RP, even if MSMRP don’t have complete knowledge about their RP’s HIV sero-status [32]. Furthermore, MSM commonly believed that condom use with a trusted RP is unnecessary, and the belief was associated with lower intention and negative attitudes regarding condom use during anal intercourse [33]. UAI may also be seen as an expression of trust among MSM [23,40]. There is a dearth of studies that explore the relationship between dyadic trust within MSM couples and their condom use. Furthermore, affectionate feeling toward a sex partner was associated with UAI among MSM [34–36]. Some MSM might express their deep emotional involvement [37], love, intimacy, and mutual commitment [30] to their RP through UAI, and believe that condom use would reduce intimacy [29]. There is a dearth of quantitative studies investigating the relationship between intimacy and UAI within MSM couples [32,33]. One study found that deeper emotional involvement was associated with perceived low risk for having UAI with RP [32].

Individual-level factors are also important. There is a lack of studies [23] investigating associations between cognitive factors (e.g. perceptions on condom use) and UAI with RP among MSMRP. It is useful to provide a theoretical framework to guide interpretation of research results. It is also important to investigate the applicability of behavioral health theories in explaining UAI with RP among MSMRP, as health-related interventions involving such theories are more effective than non-theory-based ones [41]. In this study, the Theory of Planned Behavior (TPB) [42] was used for such purposes. It has been commonly used to investigate sexual risk behaviors among MSM. The theory specifies that positive and negative attitudes, subjective norm, and perceived behavioral control predict behavioral intention, which in turn predicts the actual behavior. Mental health status is another type of individual-level factors. It is found that MSM have higher prevalence of depression than males of the general population [43]. In literature, depression was associated with UAI and higher number of male sex partners among MSM in the U.S. [38,39] and in Australia [36]. However, among HIV positive MSM in the U.S., findings on associations between anxiety and sexual risk behaviors were mixed [44]. In China, one brief report that was published in Chinese showed that depression and anxiety symptoms were positively associated with UAI among MSM in general [45].

Based on the aforementioned information, this study investigated prevalence of UAI with RP in the last three months among MSMRP in Beijing and Chengdu, China. Associated factors investigated in this study included socio-demographic characteristics, inter-personal factors (duration of relationship with RP, dyadic trust and intimacy within the relationship with RP), mental health status (depression and anxiety symptoms), and cognitive factors (perceptions on condom use based on the TPB).

## Materials and Methods

### Participants

Inclusion criteria were: 1) Chinese men of age 18 years old and above, 2) men who had anal intercourse with at least one man in the last three months, and 3) men who had at least one RP in the last three months. Those who self-reported to be HIV positive were excluded as we expected that different factors would be involved. Subgroup analysis would be required if the study had included both HIV positive and HIV negative MSM, but the sample size would be too small due to limitation in resources. We therefore only included HIV negative MSM. In this study, RPs were defined as those who were the participants’ boyfriends or those who were having a stable sexual relationship and did not involve money transaction for sex [11,46]. In cases when there were more than one RP during the last three months, participants referred to the one with whom the most recent episode of anal sex occurred.

### Data collection procedure

Publicity information about participant recruitment was disseminated through some websites and venues (e.g. bars, parks, clubs, and sauna) frequently visited by MSM. Referrals were further made by peers and participants. Interested prospective participants were invited to visit a local non-governmental organization (NGO) where trained workers briefed them about the study. Participants were informed that refusals would not affect their right to use services and they could quit the interview at any time without being questioned. During May to July 2012, a total of 343 eligible MSM was invited to join the study; 36 (10.5%) declined to participate in the study; 307 (89.5%) provided written informed consent and all of them completed the face-to-face interviews (151 in Beijing, 156 in Chengdu). The anonymous structured questionnaire took about 30 minutes to complete. The ethics approval was obtained from the Survey and Behavioural Research Ethics Committee, the Chinese University of Hong Kong. Signed informed consent was obtained from each participant in the first encounter.

### Measures

Socio-demographic characteristics of the MSM and their RP were recorded, including age, local residency (hukou), duration of stay in the Chengdu or Beijing, the highest educational level attained and personal monthly income. Participants were asked whether they had had UAI with their RP in the last three months and in the last episode of anal intercourse with the RP.

Three inter-personal variables were assessed. 1) Duration of engagement in sexual relationship with the RP was measured by a single item: “How long have you been engaged in stable sexual relationship with your regular male partner?” 2) The 8-item Dyadic Trust Scale assessed two dimensions of interpersonal trust: perceived benevolence and honesty of one’s partner [47]. It has been used in some studies targeting heterosexual couples [47,48]. A study found a significant association between the Dyadic Trust Scale and condom use among heterosexual couples [49]. In this study, this instrument was translated into Chinese by a panel of one bilingual psychologist and two bilingual epidemiologists and was back translated into English by another two bilingual epidemiologists who were blinded to the original scale. Minor changes were made. The Cronbach’s alpha of the scale was 0.81. One factor was identified by exploratory factor analysis (EFA), explaining 59.7% of the total variance. 3) The 17-item Intimacy Scale was developed by Walker and Thompson [50]. It has been widely used to assess degree of intimacy between individuals of heterosexual couples [51–54]. To our knowledge, no study has applied the scale to studies targeting MSM. The Scale [55] was also translated and modified slightly by the panel. The Cronbach’s alpha was 0.95; one factor was identified by EFA, explaining 57.7% of the total variance (Table 2).

Probable depression was measured by the Center for Epidemiologic Studies Short Depression Scale (CES-D-10), which has been widely used in studies targeting MSM in Canada [56], Russia [57], the U.S. [58], and China [45]. Scores ≥10 indicated possession of clinically significant depression symptoms (range = 0–30) [45]. In this study, one factor was identified by EFA, explaining 73.7% of the total variance (Cronbach’s alpha = 0.845). Anxiety symptoms were measured by the 7-item Generalized Anxiety Disorder Scale (GAD-7) [59]. The Chinese version had been used in a study involving MSM in Beijing [45]. A cut-off score of 15 has been recommended to define severe anxiety [59]. In this study, one factor was identified by EFA, explaining 71.8% of the total variance (Table 2; Cronbach’s alpha was 0.917).

Five constructs were derived from the TPB to assess cognitions on condom use. 1) The Positive Attitude Scale was formed by summing up the individual scores of four items (Cronbach’s alpha = 0.833). 2) The Negative Attitude Scale was formed by summing up the individual scores of two items (Cronbach’s alpha = 0.834). 3) Subjective norm was assessed by a single item “MSM do not usually use condoms during anal intercourse with RP”. 4) The Perceived Behavioral Control Scale was constructed by summing up two individual item scores (Cronbach’s alpha = 0.657). 5) Behavioral intention was measured by agreement with a single item: “You intend to use condoms with your RP every time during anal intercourse in the next three months”.

Items of the aforementioned scales are listed in tables in S1 Table.

### Statistical analysis

Prevalence of UAI with the RP in the last three months among MSMRP was presented. Univariate analysis was performed to determine factors (background variables, interpersonal variables, mental health status, and cognitive variables) associated with UAI with RP in the last three months. Variables obtaining p<.1 from such univariate analysis were used as candidates for fitting a multiple forward stepwise logistic regression model (entry p = 0.10; removal p = 0.20), and respective odds ratios (OR) were estimated from that model. Similar approaches have been used in a number of published studies [60,61]. SPSS version 17.0 was used for data analysis and p value <.05 was taken as statistically significant.

## Results

### Background characteristics of the participants and their RP

Over half of the participants were younger than 30 years old (64.5%), were non-local residents of Beijing or Chengdu (57.7%), had stayed in Beijing or Chengdu for longer than two years (72.3%), had attended a college or an university (63.2%), and had had a monthly personal income of >3000 RMB (483 USD) (53.0%). The participants’ RP had similar profiles (Table 1).

**Table 1:** Background characteristics and sexual behaviors of the participants and their RP (N = 307).

	n	%
**Socio-demographic of participants**		
Age group		
18–30	198	64.5
31–40	65	21.2
>40	44	14.3
Local resident of the city		
Yes	130	42.3
No	177	57.7
Duration of stay in the city		
≤2 years	85	27.7
>2 years	222	72.3
Highest education level attained		
Senior high or below	113	36.8
College or above	194	63.2
Monthly personal income level (RMB)		
≤3000	144	47.0
3001–5000	99	32.2
>5000	64	20.8
**Socio-demographic of their regular partner (RP** ^**a**^ **)**		
Age group		
18–30	190	61.9
31–40	77	25.1
>40	40	13.0
Local resident of the city		
Yes	133	43.3
No	174	56.7
Duration of stay in the city		
≤ 2 years	97	31.6
>2 years	210	68.4
Highest education level attained		
Senior high or below	115	37.5
College or above	192	62.5
Monthly personal income (RMB)		
≤3000	138	44.9
3001–5000	93	30.3
>5000	76	24.8
**Sexual behaviors**		
UAI with RP in the last 3 months		
Yes	161	52.4
No	146	47.6
UAI with RP in the last episode of anal intercourse		
Yes	78	25.4
No	229	74.6

^a^ For participants having multiple RPs in the last three months, they were asked to provide information of whom they recently had anal intercourse with.

### UAI with the RP

The prevalence of UAI with RP in the last three months and in the last episode of anal intercourse were 52.4% (95% CI: 46.8%-58.0%) and 25.4% (95% CI: 19.8%-31.0%), respectively. (Table 1)

### Inter-personal factors

About half (51.1%) of the participants had engaged in a sexual relationship with the RP for less than one year. The mean scores were 38.3 (SD = 10.2) for the Dyadic Trust Scale and 90.2 (SD = 20.5) for the Intimacy Scale (Table 2). Prevalence of agreement (somewhat agree/agree/strongly agree) for individual items of the Dyadic Trust Scale ranged from 18.0% to 61.6%; prevalence of participants providing responses of the Intimacy Scale reflecting an intimate relationship with the RP ranged from 55.0% to 82.0%. The results are shown in the S1 Table.

**Table 2:** Inter-personal characteristics, mental health status and cognitions related to condom use of the participants (N = 307).

	n	%	Mean	SD
**Inter-personal characteristics**				
Duration of the sexual relationship with your RP				
<1 year	157	51.1		
1–2 years	52	17.0		
>2 years	98	31.9		
Dyadic Trust Scale			38.3	10.2
Intimacy Scale			90.2	20.5
**Mental health status**				
Depression symptoms				
No clinical depression symptoms (CES-D-10 score <10)	197	64.2		
Presence of clinical depression symptoms (CES-D-10 score ≥ 10)	110	35.8		
Anxiety				
Minimal anxiety to moderate anxiety (GAD-7 score < 15)	218	71.1		
Severe anxiety (GAD-7 score ≥ 15)	89	28.9		
**Cognitions related to condom use**				
Positive Attitudes Scale			10.7	2.0
Negative Attitudes Scale			2.9	1.3
Subjective norm				
MSM do not usually use condoms during anal intercourse with RP				
Agree	87	28.3		
Disagree	161	52.4		
Uncertain	59	19.2		
Perceived Behavior Control Scale			5.4	1.0
Behavioral intention				
You intend to use condom with your RP every time during anal intercourse in the next three months				
Agree	230	74.9		
Disagree	25	8.2		
Uncertain	52	16.9		

### Mental health status

Of the participants, 35.8% scored 10 or above in the CES-D-10 (probable depression), while 28.9% scored 15 or above in GAD-7 (probable cases of severe anxiety; see Table 2).

### Perceptions on condom use derived from the TPB

With respect to positive attitudes toward condom use, a majority believed that they and their RP would feel safer when condoms were used during anal intercourse (82.7% and 76.5%), and that condoms should be used during every episode of anal intercourse (77.5% and 68.1%). Regarding negative attitudes toward condom use, some participants indicated that he and his RP believed that condom use implies mistrust (13.7% and 11.4%). For subjective norm on condom use, 28.3% agreed that MSM do not usually use condoms during anal intercourse with RP. For perceived behavioral control over condom use with RP, a majority perceived that they and their RP could persuade each other to use condoms (77.2% and 77.2%, respectively) (S1 Table). As for behavioral intention, 74.9% of the participants intended to use condom with their RP every time during anal intercourse in the next three months (Table 2).

### Univariate analysis of factors associated with UAI with the RP in the last three months

The associations between background characteristics of the participants and their RP and UAI with RP in the last 3 months were not of statistical significance (Table 3).

**Table 3:** Association between background characteristics of the participants and their RP and UAI with RP in the last 3 months (N = 307).

	n	Percentage	Univariate logistic regression
			OR
**Socio-demographic of participants**			
Age group			
18–30	198	56.1	1.0
31–40	65	44.6	0.83(0.45–1.53)
>40	44	47.7	0.82(0.34–1.98)
Local resident			
Yes	130	48.5	1.0
No	177	55.4	1.34(0.82–2.17)
Length of stay in the city			
≤2 years	85	55.3	1.0
>2 years	222	51.4	0.99(0.58–1.67)
Highest education level attained			
Senior high or below	113	51.5	1.0
College and above	194	52.1	1.43(0.07–27.34)
Monthly personal income level (RMB)			
≤3000	144	55.6	1.0
3001–5000	99	49.5	0.82(0.47–1.44)
>5000	64	50.0	0.93(0.47–1.84)
**Socio-demographic of their regular partner (RP** ^**a**^ **)**			
Age group			
18–30	190	53.7	1.0
31–40	77	42.9	0.85(0.46–1.56)
>40	40	45.0	0.85(0.35–2.04)
Local resident			
Yes	133	50.4	1.0
No	174	49.4	0.83(0.51–1.36)
Length of stay in the city			
≤2 years	97	55.3	1.0
>2 years	210	51.4	0.99(0.58–1.67)
Highest education level attained			
Senior high or below	115	50.5	1.0
College and above	192	54.1	1.15(0.72–1.84)
Monthly personal income level (RMB)			
≤3000	138	50.0	1.0
3001–5000	93	50.5	1.04(0.58–1.85)
>5000	76	48.7	0.93(0.71–1.21)

OR: odds ratios

^a^ For participants having multiple RPs in the last three months, they were asked to provide information of whom they recently had anal intercourse with.

Regarding inter-personal factors, higher scores of the Dyadic Trust Scale (OR: 1.06, 95% CI: 1.03–1.08) and the Intimacy Scale (OR: 1.03, 95% CI: 1.02–1.04) were positively associated with (risk factors of) UAI with the RP in the last three months. Duration of the sexual relationship with the RP was not of statistical significance.

With respect to individual-level variables, presence of clinical depression symptoms (OR: 1.58, 95% CI: 1.01–2.47) and probable severe anxiety (OR: 1.72, 95% CI: 1.07–2.77) were both positively associated with (risk factors of) UAI with the RP in the last three months. Regarding the four perception variables related to the TPB, higher scores of the Positive Attitudes Scale (OR: 0.58, 95%CI: 0.48–0.70) and the Perceived Behavioral Control Scale (OR: 0.71, 95% CI: 0.56–0.90), and behavioral intention to use condom with their RP every time during anal intercourse in the next three months (OR: 0.08, 95%CI: 0.04–0.17) were negatively associated with (protective factors of) UAI with RP in the last three months, while the perception that MSM do not usually use condoms during anal intercourse with RP (subjective norm) (OR: 3.57, 95%CI: 2.04–6.25) was positively associated with (a risk factor of) UAI with RP in the last three months. The positive association between UAI with the RP in the last three months and the Negative Attitudes Scale was of marginal significance, having p value between. 05 and. 10 (OR: 1.17, 95% CI: 0.98–1.39). (Table 4).

**Table 4:** Associations between inter-personal variables, mental health status, cognitions related to condom use and UAI with RP in the last three months (N = 307).

	Univariate logistic regression	Forward stepwise logistic regression
	OR (95%CI)	OR (95%CI) ^a^
**Inter-personal variables**		
Duration of the sexual relationship with your RP		
<1 year	1.0	
1–2 years	1.14 (0.58–2.240	—-
>2 years	1.11 (0.85–1.44)	—-
Dyadic Trust Scale	1.06 (1.03–1.08)***	1.04 (1.01–1.07)*
Intimacy Scale	1.03 (1.02–1.04)***	1.02 (1.01–1.04)*
**Mental Health Status**		
Presence of clinical depression symptoms	1.58 (1.01–2.47)*	2.09 (1.08–3.19)*
Probable case of severe anxiety	1.72 (1.07–2.77)*	1.06 (0.92–3.00)†
**Cognitions related to condom use**		
Positive Attitudes Scale	0.58 (0.48–0.70)***	0.76(0.61–0.95)*
Negative Attitudes Scale	1.17 (0.98–1.39)†	NS
Subjective norm		
MSM do not usually use condoms during anal intercourse with RP		
Disagree/ Uncertain	1.0	1.0
Agree	3.57 (2.04–6.25)***	2.32 (1.19–4.54)*
Perceived Behavioral Control Scale	0.71 (0.56–0.90)**	0.74 (0.56–0.96)*
Behavioral intention		
You intend to use condom with your RP every time during anal intercourse in the next three months		
Disagree/ Uncertain	1.0	1.0
Agree	0.08 (0.04–0.17)**	0.21 (0.10–0.51)**

OR: odds ratios

^a^ Odds ratios estimated by fitting a multiple forward stepwise logistic regression model (entry p = 0.10; removal p = 0.20), using variables obtaining p<.1 from univariate analysis as candidates. The model did not adjust for any socio-demographic variable, as all the socio-demographic variables listed in Table 1 all showed p>.1 in the univariate analysis.

^†^ p<.1

*p<.05

** p<.01

*** p<.001

NS: not selected by the forward stepwise logistic regression

—p>.1 in univariate analysis not included in the model

### The summary stepwise logistic regression model

In the summary model, the Dyadic Trust Scale (OR: 1.04, 95% CI: 1.01–1.07), the Intimacy Scale (OR: 1.02, 95% CI: 1.01–1.04), presence of clinical depression symptoms (OR: 2.09, 95%CI: 1.19–3.70), the Positive Attitudes Scale (OR: 0.76, 95%CI: 0.61–0.95), the perception that MSM do not usually use condoms during anal intercourse with RP (subjective norm) (OR: 2.32, 95% CI: 1.19–4.54), the Perceived Behavioral Control Scale (OR: 0.74, 95% CI: 0.56–0.96) and behavioral intention to use condom with RP every time during anal intercourse in the next three months (OR: 0.14, 95%CI: 0.06–0.35) remained significantly associated with UAI with the RP in the last three months. (Table 4)

## Discussion

In this study, over half of the MSMRP had had UAI with their RP. The findings corroborated results of other studies conducted in China [26,27] and in other countries [62]. The severity of the situation needs to be understood in the worrying context of very high HIV (13.3% and 26.2%) and STD (syphilis infection: 26.4% and 28.1%) prevalence among MSM in Beijing [63] and Chengdu [64], suggesting that MSMRP are at extremely high risk of HIV/STD infection.

Since we are concerned about UAI between MSMRP and his RP, inter-personal factors related to the regular partnership are highly relevant, and such factors should be considered when designing HIV/STD interventions targeting MSMRP. We observed high level of dyadic trust between the participants and their RP; the majority of the participants believed that their RP were honest to them and cared about their wellbeing. The level of intimacy between MSMRP and their RP was also high as the majority of the participants showed strong affection toward their RP and were willing to share thoughts and feelings with their RP. Importantly, both trust and intimacy were significantly and positively associated with UAI with RP and hence require some attention. MSMRP may not necessarily know about the sexual history and HIV status of their RP. High level of trust may lead MSMRP to believe that their RP are risk free and hence to underestimate the risk of HIV transmission involved when having UAI with the RP [33]. The chance of having UAI would then increase. Also, some MSM may further use UAI as an expression of intimacy [37], which may partially explain the observed positive association between intimacy and UAI.

In terms of the findings’ implications onto designing of HIV prevention interventions, first, risk perception about HIV/STD transmission among MSMRP needs to be heightened. MSMRP should be reminded about HIV prevention programs on basic epidemiology of HIV transmission among MSM in China, which indicates that the prevalence of multiple partnerships among RP of MSMRP is high [18], and that UAI with RP is a strong risk factor of HIV transmission among MSMRP [26]. They should also be reminded that they might not know about the complete sexual history and HIV status of their RP and cannot eliminate existing risk of HIV transmission. It should be further clarified that trust and intimacy might have become obstacles instead of facilitators in preventing HIV infection. Suggestions may be made that HIV/STD infection would destroy the dyadic trust and intimacy that they value [32], while condom use may alternatively be a way to uphold trust and intimacy. Male same-sex couples should be encouraged to discuss openly and explicitly about meanings of trust/intimacy of the relationship with respect to condom use. Since such discussions need to involve the couple, couple-based counseling is warranted. Such interventions need to build up communication skills and to handle discrepancy in willingness to use condoms and potential emotions and conflicts that arise during the communication process.

Furthermore, future research may be conducted to investigate the applicability of the concept of sexual agreement as an HIV prevention effort among MSMRP in China [65,66]. Sexual agreement is defined as a mutual and explicit agreement made by a MSM couple about acceptability and conditions of having sex with someone outside the regular partnership. The common forms of sexual agreement include an agreement that there would be no sexual encounters outside the dyad (known as a closed agreement), an agreement that sex with other sex partners would be allowed but only under certain conditions such as consistent condom use (known as an in-between agreement), and an agreement that both of them would be allowed to have sex freely with other sex partners (known as an open agreement) [65,66]. Importantly, research showed that existence of a closed agreement or an in-between agreement conditional on consistent condom use with other sex partners would reduce UAI among MSMRP [65]. Open discussion about sexual agreement and condom use could potentially overcome issues rising from the association between trust and intimacy and UAI with RP. Although a sexual agreement could be breached, it may reduce the chance of underestimating risk involved in UAI with each other due to the trust that the partner is risk free and may reduce potential fear to undermine intimacy with condom use. It may also allow MSMRP to understand the sexual history of their RP better. HIV prevention programs encouraging establishment of sexual agreement are potentially feasible as many MSM couples (66% to 100%) in western countries, such as U.S., Australia and Netherland, have made such sexual agreements [67–69]. To our knowledge, there is no published study reporting prevalence of sexual agreements among MSMRP in China. Pilot studies and randomized controlled studies are required to test feasibility and efficacy of this novel approach.

Besides, there is a dearth of studies investigating mental health problems among male same-sex couples and the impact of such problems on their HIV-related risk behaviors. About one-third or more of the sampled MSMRP presented clinical depression or severe anxiety symptoms, and probable depression was significantly and positively associated with UAI with RP. Depression and anxiety undermine individuals’ intention to take up protective measures such as condoms [70]. Mental health problems may result in poor support-seeking; UAI may also be used as a negative coping response to mental health problems [43,71]. Furthermore, it is known that MSM are less likely than heterosexual males to seek professional services to treat their mental health problems [70], possibly due to perceived stigma and self-stigma from their sexual orientation [72]. Our data hence suggest that there is a strong demand for mental health support services that are friendly to MSM. Such services should also become a regular and integrated part of HIV prevention efforts targeting MSMRP.

We pointed out that mental health problems of MSMRP were associated with UAI with their RP. Previous studies have further shown that mental health problems of the MSM and their RP are inter-related [73]. It is a limitation of this study that we did not assess the mental health status of the RP as such assessment through data reported by the participant may not be reliable. However, we expect high prevalence of concordant depression and anxiety within the MSM couples as previous studies have consistently found among MSM in general [74]. Having both of the couple feeling depressed would add difficulties to their communication with each other about issues related to condom use, and to overcome obstacles in condoms use due to concerns of trust and intimacy [72]. Couple-based mental health support and counseling services are warranted. Whilst such services are widely available for heterosexual couples, they may be unavailable for male same-sex couples, despite a large demand. In most countries, same-sex marriage is illegal [75]. Together with strong social stigma toward MSM, same-sex couples suffering from mental health problems are unlikely to seek professional services together to improve their situation, although such couple-based counseling would be potentially useful. Such obstacles need to be overcome, filling an important service gap.

Perceptions toward condom use based on the TPB were associated with UAI with the RP in the last three months. Contents of HIV prevention programs promoting condom use with RP among MSMRP may make references to such findings. First, it is important to promote positive attitudes toward condom use. One possibility is to promote the concept that consistent condom use with the RP implies care for each other and that it is important to feel safe from consistent condom use. Second, health care workers need to consider the current subjective norm among MSMRP that it is unnecessary to use condoms with the RP, as shown by our data. Testimonials made by influential MSM peers such as NGO leaders and homosexual celebrities might be useful in creating a new subjective norm supporting consistent condom use with the RP. Third, we found that perceived behavioral control over condom use with RP was negatively associated with UAI with RP. As mentioned, couple-based counseling should be provided to MSMRP to enhance their communication skills and to obtain support from their RP with respect to consistent condom use during anal intercourse so as to increase behavioral control. Role plays and skill training have also been found effective in improving behavioral control over condom use among MSM [76]. It is encouraging that over 70% of the MSMRP intended to use condoms consistently with their RP in the next three months. There is hence room for improvement.

Some comments need to be made concerning our findings and suggestions for future HIV interventions. First, the multivariate summary model suggested that trust and intimacy, depression and perceptions toward condom use (positive attitudes, subjective norms, and perceived control over condom use of the TPB) were all independently associated with UAI with RP. Collinearity was hence not an issue in the modeling. It implies that factors regarding relationship, mental health problems, and perceptions toward condom use should all be integrated in relevant HIV prevention interventions. Second, we understand that the results were associations in nature rather than causal. Therefore, we suggest further research to test the feasibility and efficacy of an integrated HIV prevention program that take into consideration the suggested components targeting MSMRP. Specifically, it may include activities attempting to change attitudes and to increase behavioral control related to condom use, and couple-based counseling to deal with issues of mental health problems such as depression, communication on condom use that might include introducing the aforementioned concept of sexual agreement if it is found acceptable by the client, and to reduce obstacles of condom use due to concerns about trust and intimacy. Evidence from such programs is needed. Lastly, it should be kept in mind that although the literature has shown clearly that UAI with RP is a risk factor of contracting HIV among MSMRP, it is not the only risk factor. We should not give the impression that MSMRP should not trust each other and be intimate with each other. Instead, we would like them to assess their risk properly and to remind them that trust and intimacy should not become hurdles against consistent condom use, which is doubtlessly the most effective and feasible means of HIV prevention [77].

This study had some limitations. First, the participants were recruited by using convenience sampling as there was no sampling frame. The results may not be representative of MSM in Beijing as some MSM were not accessible through gay venues or personal networks. Respondent-driven sampling methods, which may increase representation, was not used in this study [78]. Second, we did not record the number of participants approached, nor the number of MSM recruited but not eligible; we also did not collect information from participants who declined to participate in the study. Third, data were self-reported. Reporting bias may exist although the study was anonymous, and computer assisted methods could have been used to reduce reporting bias. Fourth, we confined our sample to HIV negative MSM as different factors of UAI would have been involved among HIV positive and among HIV negative MSM [79–81]. Ideally, we should have stratified our data analysis on UAI by subgroups according to participants’ HIV status. However, such subgroup analysis would require a much larger sample size and was not feasible due to our limited resources. Fifth, we did not ask information about multiple regular partnerships. Moreover, definition of RP was subjective, although such definitions have been used in many similar studies. Furthermore, the cognitive variables of the TPB were constructed by us, in the absence of available scales [82]. Lastly, the cross-sectional study design was unable to establish causal relationships.

## Conclusion

In sum, MSMRP were at very high risk of HIV/STD transmission. Special issues with respect to risk factors arising from the nature of the regular partnership, including dyadic trust and intimacy, need to be addressed in order to reduce UAI with the RP. We point out that the high prevalence of mental health problems further increase difficulties in HIV prevention. Future studies should look at the inter-relationship in mental health status between MSMRP and their RP and should integrate mental health support with HIV prevention. Modifications of perceptions related to condom use such as enhancing positive attitudes, creating a new supportive subjective norm, and increasing perceived behavioral control over condom use, are also suggested to be integrated in HIV interventions targeting MSMRP, a highly vulnerable but under-served population. A randomized control study to test feasibility and efficacy of the suggested integrated couple-based intervention is warranted.

## Supporting Information

S1 Table Item responses of the scales used in the study (N = 307). (DOC)
