# Supplementary material for: Prevalence and Associated Factors of Unprotected Anal Intercourse with Regular Male Sex Partners among HIV Negative Men Who Have Sex with Men in China: A Cross-Sectional Survey
Source: PLoS One. 2015 Mar 27;10(3):e0119977. doi: 10.1371/journal.pone.0119977 (PMC4376721; doi:10.1371/journal.pone.0119977)
Supplement: S1 Table — (DOC) [file pone.0119977.s001.doc]

S1 Table: Item responses of the scales used in the study (N=307)

|  | % |
| --- | --- |
| **Dyadic Trust Scale** (% somewhat agree/agree/strongly agree) |  |
| My RP is primarily interested in his own welfare | 18.0 |
| There are times when my RP cannot be trusted | 23.1 |
| My partner is perfectly honest and truthful with me | 51.9 |
| I feel that I can trust my RP completely | 53.2 |
| My RP is truly sincere in his promises | 53.8 |
| I feel that my RP does not show me enough consideration | 30.8 |
| My RP treats me fairly and justly | 50.0 |
| I feel that my RP can be counted on to help me | 61.6 |
| **Intimacy Scale** |  |
| How often do you have these feelings in the relationship with your RP  (% frequently/almost always/always) |  |
| We want to spend time together | 72.9 |
| My RP shows that he loves you | 69.4 |
| We are honest with each other | 68.1 |
| We can accept each other’s criticism of our faults and mistakes | 73.5 |
| We like each other | 82.0 |
| We respect each other | 79.4 |
| Our lives are better because of each other | 60.3 |
| We enjoy the relationship | 68.6 |
| My RP cares about the way I feel | 75.3 |
| We feel like we are a unit | 71.6 |
| There’s a great amount of unselfishness in our relationship | 59.8 |
| My RP always thinks of my best interest | 50.0 |
| I am lucky to have him in my life | 77.6 |
| My RP always makes me feel better | 72.8 |
| My RP is important to me | 68.3 |
| We love each other | 61.8 |
| I’m sure of this relationship | 69.8 |
| **Positive Attitudes Scale** |  |
| You would feel safer if you use condom during anal intercourses with your RP |  |
| Agree | 82.7 |
| Disagree | 5.9 |
| Uncertain | 11.4 |
| Your RP would feel safer if your RP uses condoms during anal intercourse with you |  |
| Agree | 76.5 |
| Disagree | 7.5 |
| Uncertain | 16.0 |
| You believe that condoms should be used during every anal intercourse with your RP |  |
| Agree | 77.5 |
| Disagree | 7.5 |
| Uncertain | 15.0 |
| Your RP believes that condoms should be used during every anal intercourse with you |  |
| Agree | 68.1 |
| Disagree | 10.7 |
| Uncertain | 21.2 |
| **Negative Attitudes Scale** |  |
| You believe that condom use implies distrust |  |
| Agree | 13.7 |
| Disagree | 70.0 |
| Uncertain | 16.3 |
| Your RP will believes that condom use implies distrust |  |
| Agree | 11.4 |
| Disagree | 68.1 |
| Uncertain | 20.5 |
| **Perceived Behavioral Control Scale** |  |
| Your RP can persuade you to use condom if you don’t want to. |  |
| Agree | 77.2 |
| Disagree | 8.5 |
| Uncertain | 14.3 |
| You can persuade your RP to use condom if he doesn’t want to. |  |
| Agree | 77.2 |
| Disagree | 7.5 |
| Uncertain | 15.3 |
